# Analysis of Endothelin-1 Concentrations in Individuals with Periodontitis

**DOI:** 10.1038/s41598-020-58585-4

**Published:** 2020-02-03

**Authors:** Gaetano Isola, Alessandro Polizzi, Angela Alibrandi, Francesco Indelicato, Sebastiano Ferlito

**Affiliations:** 1 Department of General Surgery and Surgical-Medical Specialties, School of Dentistry, University of Catania, Via S. Sofia 78, 95123 Catania, Italy; 2 Department of Economics, Unit of Statistical and Mathematical Sciences, University of Messina, Messina, Italy

**Keywords:** Medical research, Oral diseases

## Abstract

Endothelin 1 (ET-1) has been shown to have a key role in homeostasis and function of endothelium and maybe fundamental in the relationship between coronary heart disease (CHD) and periodontitis. In this trial, we assessed the influence on serum and salivary ET-1 levels of gingival health, CHD, periodontitis, or a combination of periodontitis-CHD. Clinical and periodontal parameters, were collected from periodontitis patients (n = 34), CHD patients (n = 34), periodontitis + CHD patients (n = 34), and from healthy patients (n = 34) together with saliva and serum samples. The median concentrations of salivary and serum ET-1 were significantly higher in the CHD patients [serum: 1.4(1.1–1.6) pg/ml; saliva 1.2 (0.9–1.6) µmol/g, p < 0.01] and in the periodontitis + CHD patients [serum: 1.7 (1.2–21.8) pg/ml; salivary 1.4(1–1.6) µmol/g, p < 0.001] respect to periodontitis and control patients. Through a univariate regression analysis, c-reactive protein (CRP) and CHD (both p < 0.001) and periodontitis (p = 0.029) were statistically correlated with ET-1 in serum. The multivariate regression analysis demonstrated that only CRP was the statistically predictor of ET-1 in serum(p < 0.001). The multivariate regression analysis in saliva demonstrated that, regarding ET-1 levels the only predictor were CRP (p < 0.001) and total cholesterol (p = 0.042). The present study evidenced that subjects with CHD and periodontitis plus CHD had higher serum and salivary levels of ET-1 compared to subjects with periodontitis and healthy controls. Moreover, only CRP remained a major predictor of increased ET-1 concentrations in both serum and saliva.

## Introduction

Periodontitis is a common oral inflammatory multifactorial disease that determines the disruption of the periodontium and the tissues that supporting the tooth, such as bone and cementum main caused by oral bacteria, that determines finally loss of the tooth^1^. Almost of adults in the USA present periodontal disease forms and nearly ten percent of the population in worldwide express severe type of periodontal disease^2,3^.

More recently, observational reports have shown an correlation between periodontal disease and cardiovascular disease, including coronary heart disease (CHD), stroke, and endothelial dysfunction^4,5^. Moreover, some studies demonstrated a specifi correlation among periodontal disease and augmented risk of stroke and, in general, CHD^6–8^.

The pathogenesis of periodontal disease includes inflammatory and bacteria responses which may determines an increased host response subsequent to the presence of pathogenic oral biofilm in gingival tissues^9^. More specifically, periodontal disease has been correlated an increase of levels of some systemic inflammatory mediators in serum, such as interleukin 1 (IL-1), IL-6, prostaglandin, and C-reactive protein (CRP)^10^.

Endothelin (ET)-1 is one of the most frequent findings of ET in the human body expressed within tissues during the progression of inflammation^11^. It was demonstrated that the ET-1, secreted by endothelial cells after exposure to pathogenic bacteria, represents a potent mediator of vascular inflammation and a vasoconstrictor^12^.

On this regard, it has been shown that several proinflammatory cytokines, including IL-1, -6, and -8, have been reported to upregulate the secretion of ET-1^13^. The expression of ET-1 was strongly associated in the gingival tissue and endothelial cells during periodontitis^14^. More specifically, a clinical study found that, in gingival crevicular fluid, the ET-1 level increased with the progression of the periodontitis, and also that ET-1 was involved in the regulation of IL-1b expression in gingival tissues^15,16^.

During the last few decades, several evidences have analysed the association between periodontal disease endothelial dysfunction, and increased risk of CHD and CVD^17,18^. More specifically, has been supposed that the inflammatory mediators that are present and released during the active phase of periodontal disease such as CRP, interleukins, prostaglandins, and metalloproteases, can negatively influence the release of Nitric Oxide (NO)^19^. The alterated production of NO can affect the vascular endothelial cells which in turn regulates vascular tone, and, finally endothelial dysfunction and augmented risk of CVD^20,21^. For these causes, there is growing interest aimed to investigate in some other oral mediatoris that can regulate and impact the subclinical endothelial dysfunctions as an ealry sign of augmented risk of CHD and CVD.

An association between high serum ET-1 and CRP levels and endothelial damage was recently reported^22^. A cross-sectional study demonstrated that periodontitis is associated, in a dose-dependent manner, with impaired serum ET-1 levels in patients^23^.

The production of NO at local level has been shown to be fundamental in the aetiology and progression of periodontitis. The increment and decrement in the salivary NO metabolites production in gingival tissue against periodontal bacteria during periodontitis have been reported to be correlated with impaired endothelium-dependent vasodilatation^24,25^. More specifically, it has been shown that ET-1 acts as a competitive inhibitor of the NO synthase and that increased serum ET-1 levels have been reported in some metabolic disorders including periodontitis^26^.

To date, there are no studies that evaluate both salivary and serum ET-1 levels during periodontitis. The aims of this preliminary study were to consider a possible impact of periodontitis, CHD, or a combination of both diseases on serum and salivary ET-1 levels. Moreover, it was assessed the possible association between both saliva and serum ET-1 levels and if serum CRP mediated the association between salivary or serum ET-1 levels.

## Materials and Methods

### Study design

For the present study, 348 healthy controls and patients with periodontitis or CHD were selected among those who attended the Department of Periodontology, School of Dentistry, Messina, Italy, from March 2016 to October 2018. Groups were selected on a prespecified age range (40–60 years old) and on sex so that similar proportion to the cases fall into the categories defined by the selection variable. 50% of the cases and controls were males aged 46–58 years.

The study was performed by the Declaration of Helsinki, revised in 2016 on medical research. Ethical approval was obtained by the IRB of the University of Messina (#16012). The study was registered at clinicaltrials.gov (NCT04152005). Written informed consent was obtained from each enrolled subject (both for patients and healthy controls) about the study characteristics and possible risks of the study. This study followed the STROBE guidelines for the strengthening of reporting of observational studies^27^.

Inclusion criteria for the periodontitis group were: (1) presence of at least 16 teeth, (2) a minimum of 40% of sites with clinical attachment level (CAL) ≥2 mm and probing depth (PD) ≥4 mm^28^; (3) presence of at least one site for each quadrant with ≥2 mm of crestal alveolar bone loss verified on digital periapical radiographs; (4) presence of ≥40% sites with bleeding on probing (BOP)^29^. Healthy individuals presented no systemic disease, ≤10% sites with BOP, no sites with PD ≥4 mm or CAL ≥4 mm, no sites with BOP^29^ or radiographic signs of bone loss.

Inclusion criteria for the CHD group were: at least ≥18 years old with a diagnosis of CVD, ≥50% of stenosis of at least one coronary artery verified by coronary angiography or a coronary artery bypass surgery, or past or current percutaneous coronary intervention^30^. Information on previous medical conditions, cardiovascular risk factors, medications, electrocardiography, echocardiography, and coronary angiogram results were collected. In all patients, the diagnosis of CHD was performed by the same operator (SF) from medical record information. For the periodontitis + CHD subjects the inclusion criteria were based on the same criteria of the single periodontitis and CHD groups but combined.

For all enrolled subjects, were excluded if presented (1) consumption of contraceptive drugs; (2) consumption of antibiotics, anti-inflammatory or immunosuppressive drugs during the recent 3 months previous the trial; (3) presence of gestation or suction; (4) intake of alcohols; (5) anesthetic allergy; (6) intake of Nifedipine, Hydantoin or Cyclosporin A drugs; (7) periodontal therapy in the last 3 months before the study baseline.

Then, 212 subjects were left out from the study analysed patients because not encounter the inclusion criteria (n = 147), failed to join in the study (n = 41), or were los at the first assessment (n = 24). For these reasons, for the present study, 34 healthy subjects, 34 periodontitis patients, 34 CHD patients, and 34 patients with a combination of both disease (periodontitis plus CHD), and were enrolled in the end (Supplementary Fig. 1).

In each patient, every demographic characteristic (such as educational level) and demographic indices such as age, sex, body mass index (BMI), diabetes, hypertension, dyslipidemia, and other systemic events were recorded together with the type of drug taken. The presence of diabetes was recorded based on the patient’s medical history or on fasting blood glucose ≥126 mg/dl. The BMI was recorded by calculating the patient’s weight divided by the square of his height in kg/m^2^. All enrolled subjects were also classified on their smoking history such as active smokers, ex-smokers (patients who have not smoked for at least ≥5 years) and non-smokers.

The periodontal evaluation comprised clinical attachment loss (CAL), probing depth (PD), bleeding on probing (BOP), and plaque score (PI)^31^. CAL was verified such as PD + gingival recession using the cementoenamel junction as a reference. All periodontal index were registered, in all patients, by two independent calibrated examiners (a principal examiner and a control examiner), exonerated in the subsequent study steps, using a periodontal probe^‖^. The inter- and intra-examiner reliability of the outcomes PD and CAL were assessed using the Intraclass Correlation Coefficient (ICC). The inter-examiner reliability resulted in an agreement for PD (ICC = 0.821) and CAL (ICC = 0.823) denoting a reasonable degree of reliability for both parameters. The intra-examiner reliability of PD and CAL was performed only on 28 selected patients (7 patients per group chosen randomly) for both examiners. The intra-examiner reliability for the first examiner resulted in an agreement for PD (ICC = 0.855) and CAL (ICC = 0.831); for the second examiner, it resulted in an agreement for PD (ICC = 0.811) and CAL (ICC = 0.19) denoting a reasonable degree of reliability for both parameters. All periodontal index were registered, in all patients, at 6 sites in each tooth in all tooth element that were present.

A power analysis was executed in order to evaluate sample size needed for the study. The sample size was determined pondering 4 groups, an effect size of 0.30 for ET-1 (that represented the primary outcome variable), an expected standard deviation of 1.5^23^ a 2-sided significance level of 0.05 and a power of 80%. It was established that around 32 patients per group would be required. A total number of 128 patients were needed to ensure at least a power level of 80%. Were enrolled one hundred and thirty-nine patients so that the study power was 84%. Power and sample size calculation was performed using statistical software**.

### Evaluation of salivary and serum ET-1

All serum and saliva samples were collected on an in all patients enrolled between 8:00 and 10:00 a.m., before the periodontal examination, on the same day by the same examiner (FI). All enrolled subjects were requested to refrain from drinking, eating, chewing, brushing their teeth or other oral hygiene maneuvers in the 12 hours preceding the sampling of serum and saliva.

For the collection of serum, a venous blood sample was taken which, after the collection, was immediately cooled with ice and centrifuged at 4 °C (800 × g for 10 min). For the collection of saliva samples, the enrolled patients were asked to moisten by chewing a cotton roll for two minutes using the salivette method^¶^. Subsequently, the saliva sample in each patient was immediately centrifuged at 4 °C (1000 × g for 2 min). Both saliva and serum samples collected were stored at −20 °C for subsequent analysis.

Enzyme-linked immunosorbent assays (R&D Systems, Minneapolis, MN, and Sigma-Aldrich, St Louis, MO) were used to detect serum and salivary concentrations of ET-1, following the manufacturers’ instructions. Levels of hs-CRP were calculated using a nephelometric assay kit. Levels of hs-CRP >3 mg/L were related to an augmented CVD risk. Routine methods were applied to assess glucose and plasma lipids levels.

### Statistical analysis

Median, 25%, and 75% percentile were used to express numerical variables while number and % were used to express categorical variables. Most of the variables analyzed (e.g. triglycerides, fasting glucose and all periodontal index) not had normal distribution such as confirmed by Kolmogorov–Smirnov test. Only age, BMI and salivary and serum ET-1 were normally distributed; for this reason, nonparametric tests were used to analyse all data in the present analysis^32^. More specifically, to confront all numerical variables in the 4 groups of patients, was applied the Kruskal Wallis test while the Mann Whitney test was applied to obtain the two-by-two comparisons. Bonferroni’s correction was applied for numerous evaluations; the α level of 0.050 was split by the potential comparisons (n = 6), and the adjusted significance level equalled 0.008 (0.050/6).

The p-trend analysis for salivary and serum and ET-1 levels was obtained using the Jonckheere-Terpstra Test to evaluate whether ET-1 levels were staitistically augmented in the four analyzed groups. To asses any significant interdependence between ET-1 in saliva and serum and hs-CRP, the Spearman correlation test was used.

Moreover, a univariate and multivariable linear regression analysis were applied in all enrolled patients to evaluate the dependence of ET-1 levels in serum and saliva (which resulted normally distributed) on possibly explicative outcomes such as sex, education, age, socioeconomic status (SES), triglycerides, total cholesterol, BMI, CRP, and CVD drugs (yes/no). In the multivariate final model, sex, age, and education SES were incorporated such as possible confounders, and tested to analyze if CHD, periodontitis,and hs-CRP influenced ET-1 in serum. For the evaluation of ET-1 in saliva, the same analysis was performed using salivary ET-1 levels as an outcome. All statistical analyses were executed using statistical software. ^†^A p-value < 0.05 was considered to be statistically significant.

## Results

The demograpghic and serological characteristics of the enrolled patients are shown in Table 1. All groups were matched for age and sex, and there did not presented any statistically differences regarding education levels, smoking, BMI, and serological features (Table 1).

**Table 1:** Individual characteristics and biochemical parameters of recruited subjects. Data is expressed as median (25th; 75th percentiles) or number with percentage. CHD, coronary heart disease; CVD, cardiovascular disease.

	Controls (N = 34)	Periodontitis (N = 34)	CHD (N = 34)	Periodontitis + CHD (N = 34)
Age (years)	55 (52; 57)	54 (51; 56)	55 (51; 57)	56 (52; 58)
Gender (male/female)	17/17	18/16	16/18	17/17
**Education level**				
Primary school, n (%)	12 (35.2)	13 (38.2)	12 (35.2)	13 (38.2)
High school, n (%)	15 (44.1)	13 (38.2)	13 (38.2)	13 (38.2)
College/university, n (%)	7 (20.6)	8 (23.5)	9 (26.5)	7 (20.6)
Body mass index (kg/m^2^)	24.9 (21.8; 27.8)	24.4 (22.5; 26.5)	25.2 (22.7; 28.6)	25.5 (21.1; 25.2)
Fasting glucose (mg/dl)	88.1 (85.4; 92.3)	89.2 (80.1; 119.2)	89.2 (81.4; 121.5)	90.6 (86.5; 122.2)
Smokers, n (%)	4 (11.7)	4 (11.7)	3 (8.8)	5 (14.7)
Never smokers, n (%)	30 (88.2)	29 (85.2)	30 (88.2)	29 (85.2)
Past smokers, n (%)	1 (3)	1 (3)	1 (3)	2 (5.9)
Current smokers, n (%)	3 (8.8)	3 (8.8)	2 (5.9)	3 (8.8)
**Comorbidities**				
Diabetes, n (%)	—	2 (5.9)**	3 (8.8)**	3 (8.8)**
**Previous CVD**				
Atrial fibrillation, n (%)	—	—	7 (20.6)**^,§§^	9 (26.5)**^,§§^
Angina pectoris, n (%)	—	—	14 (41.2)**^,§§^	15 (44.1)**^,§§^
Stroke, n (%)	—	—	7 (20.6)**^,§§^	9 (26.5)**^,§§^
Heart failure, n (%)	—	—	5 (14.7)**^,§§^	6 (17.6)**^,§§^
**Drug treatment of CVD**				
Antihypertensive, n (%)	—	—	11 (32.3)**^,§§^	12 (35.2)**^,§§^
Statins, n (%)	—	—	12 (35.2)**^,§§^	10 (29.4)**^,§§^
Low-dose aspirin, n (%)	—	—	8 (23.5)**^,§§^	8 (23.5)**^,§§^
Beta blockers, n (%)	—	—	9 (26.5)**^,§§^	10 (29.4)**^,§§^
hs-CRP (mg/L)	2.7 (2.2; 2.8)	3.4 (2.8; 3.7)*	5.9 (5.2; 6.4)**	6.7 (5.8; 7.5)**^,§§,#^
Total cholesterol (mg/dl)	154 (125; 176)	169 (137; 177)	170 (145; 184)	175 (161; 194)
Triglycerids (mg/dl)	129 (99; 139)	109 (59; 131)	141 (122; 157)	144 (119; 166)

*p < 0.001 and **p < 0.001 significant differences vs healthy subjects calculated by the Mann Whitney test. ^§§^p < 0.001 significant differences vs periodontitis patients calculated by the Mann Whitney test. ^#^p < 0.008 significant differences vs CHD patients calculated by the Mann Whitney test.

Compared to healty controls, patients with periodontitis, CDH and a combination of periodontitis+CHD presented a higher values of hs-CRP (p < 0.001). Moreover, patients with CHD and periodontitis plus CHD presented no significant differences regarding past CVD events.

In Table 2 are represented dental characteristics of all enrolled patients patients. Compared with CHD and control patients, subjects with periodontitis and periodontitis + CHD showed higher periodontal parameters (CAL, PD, BOP, PI) and few number of teeth (p < 0.001) (Table 2).

**Table 2:** Clinical dental variables of recruited subjects. Data is expressed as median (25th; 75th percentile). CAL, clinical attachment level; PD, probing pocket depth; BOP, bleeding on probing; PI, Plaque index.

	Controls (N = 34)	Periodontitis (N = 34)	CHD (N = 34)	Periodontitis+CHD (N = 34)
N° of teeth	25 (21; 27)	19 (16; 18)**	23 (20; 24)**^,§§^	18 (13; 21)**^,##^
CAL (mm)	1.1 (0.8; 1.5)	3.7 (3.2; 4.1)**	2.1 (1.9; 2.2)**^,§§^	3.8 (3.5; 4.6)**^,##^
CAL 4–5 mm (% sites)	—	38.7 (36.3; 41.8)**	—	41.8 (36.5; 47)**^,##^
CAL ≥6 mm (% sites)	—	18.7 (18.2; 21.4)**	—	17.9 (16.1; 23.8)**^,##^
PD (mm)	1.6 (1.2; 1.8)	4.2 (3.9; 4.7)**	2 (1.7; 2.2)**^,§§^	3.9 (3.7; 4.4)**^,##^
PD 4–5 mm (% sites)	—	41.4 (40.6; 44.7)**	—	44.5 (41.3; 52.1)**^,##^
PD ≥6 mm (% sites)	—	21.3 (18.8; 23.1)**	—	23.9 (21.2; 27.6)**^,§§,##^
BOP (%)	8.1 (6.1; 9.6)	41.5 (35.4; 47.6)**	8.5 (6.7; 8.4)**^,§§^	45.5 (42.6; 51.5)**^,§§,##^
Rx alveolar bone loss (mm)	0.2 (0.1; 0.4)	2.9 (2.6; 3.5)**	0.4 (0.2; 0.5)**^,§§^	3.4 (2.2; 4.2)**^,##^
PI (%)	6.7 (5; 9.5)	36.6 (32.9; 34.6)**	12.9 (10.1; 13.4)**^,§§^	31.6 (29.1; 35.4)**^,##^

**p < 0.001 significant differences vs control subjects calculated by the Mann Whitney test. ^§§^p < 0.001 significant differences vs periodontitis patients calculated by the Mann Whitney test. ^##^p < 0.001 significant differences vs CHD patients calculated by the Mann Whitney test.

**Figure 1:**
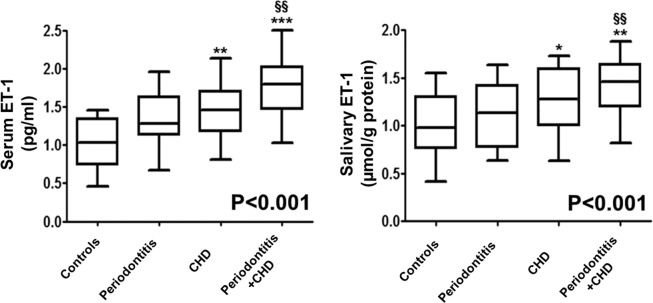
Median values (25%; 75% percentiles) of serum and salivary ET-1 levels in each group of subjects. *p < 0.05, **p < 0.01 and ***p < 0.001 significant differences vs. control subjects (derived by Kruskal Wallis test). ^§§^p < 0.01 significant differences vs periodontitis patients. P < 0.001 (obtained by Jonckheere-Terpstra test).

Figure 1 represent median (25^th^; 75^th^ percentile) values of ET-1 levels in saliva and serum of all enrolled patients. Compared to control subjects, patients with CHD (p < 0.01) and with periodontitis plus CHD (p < 0.001) had higher median concentrations of ET-1 in saliva and serum. More specifically, in comparison with periodontitis subjects, patients with periodontitis + CHD presented increased salivary and serum concentrations of ET-1 (p < 0.01) (Fig. 1). Moreover, the p-for trend analysis test evidenced that ET-1 in serum increased gradually in subjects with periodontitis, CHD, and with periodontitis + CHD (p-trend < 0.001) (Fig. 1).

**Figure 2:**
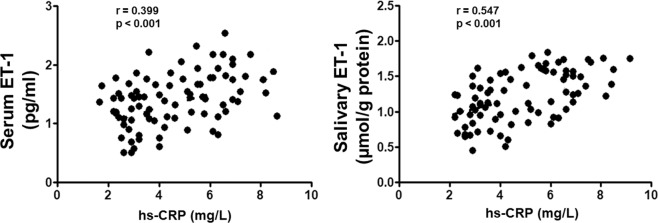
Correlation analysis of serum and salivary ET-1 levels with CRP values in all enrolled subjects.

There was no significant correlation of ET-1 levels between serum and saliva (r_s_ = 0.213, p = 0.098). Moreover, in all enrolled patients presented a positive correlation between serum/salivary ET-1 and hs-CRP levels (r_s_ = 0.399, p < 0.001)/(r_s_ = 0.547, p < 0.001) (Fig. 2).

The univariate regression analysis evidenced that there was a significant direct impact of hs-CRP on serum and salivary ET-1 (both p < 0.001). Furthermore, the adjusted multivariate linear regression analysis, aimed at assessing the possible influence of both periodontitis and CHD on serum and salivary ET-1 levels, showed that hs-CRP (p < 0.001) was the only statistically significant predictor variable for serum and ET-1.

Moreover, hs-CRP (p < 0.001) and total cholesterol (p = 0.043) were the statistically significant predictor variables for salivary ET-1 levels (Table 3).

**Table 3:** Uni and multivariate linear regression model for serum and salivary ET-1 levels in all enrolled subjects. Age was included as continuous variable. For periodontitis and CHD, controls served as reference. For gender, male served as reference. For education, primary school served as a reference.

Serum ET-1 levels	Univariate			Multivariate		
Variable	B	95% CI	*P*	B	95% CI	*P*
CHD	0.377	0.216; 0.447	**<0.001**	0.064	−0.299; 0.475	0.684
Periodontitis	0.222	0.011; 0.397	**0.029**	0.123	−0.049; 0.413	0.177
hs-CRP	0.139	0.059; 0.112	**<0.001**	0.211	0.087; 0.117	**<0.001**
Age (years)	−0.011	−0.341; 0.012	0.087	−0.045	−0.125; 0.236	0.411
Female gender	0.133	−0.055; 0.412	0.185	0.147	−0.97; 0.412	0.057
Education SES	−0.079	−0.187; 0.064	0.312	−0.078	−0.312; 0.478	0.254
Salivary ET-1 levels						
CHD	0.211	0.136; 0.654	**<0.001**	−0.058	−0.541; 0.313	0.549
Periodontitis	0.055	−0.087; 0.331	0.378	0.008	−0.184; 0.168	0.784
hs-CRP	0.074	0.038; 0.223	**<0.001**	0.074	0.038; 0.145	**<0.001**
Age (years)	−0.041	−0.031; 0.009	0.201	0.005	−0.045; 0.036	0.841
Female gender	0.055	−0.145; 0.236	0.491	0.079	−0.044; 0.315	0.231
Total Cholesterol	−0.079	−0.174; −0.012	**0.042**	−0.87	−0.058; 0.239	**0.043**
Serum ET-1	0.139	−0.035; 0.413	0.077	−0.028	−0.312; 0.174	0.553

## Discussion

This trial was aimed at evaluating the impact of conditions such as periodontal disease, CHD, or a combination of both periodontitis + CHD on ET-1 levels in serum and saliva. The present trial evidenced that the occurrence of CHD contributed to increased levels of serum and salivary ET-1 and hs-CRP levels. However, compared with periodontitis and healthy subjects, only the group of subjects with CHD and periodontitis plus CHD had significantly elevated ET-1 levels in serum and saliva, supporting the hypothesis that CHD has contributed to increased ET-1 levels in serum and saliva.

Furthermore, results of the present study show that the simultaneous presence of periodontitis in patients with CHD can determine an increased activation of ET-1 and therefore represent a subclinical stimulus for the purpose of an increase in CVD development. In accordance with the results of the present study, some reports have shown that high levels of ET-1 in serum represent real independent risk factors of CVD development and increased mortality index, possibly by inactivating NO.^33^ signaling. Specifically, it has also been shown that, in patients with atherosclerosis, high systemic levels of ET-1 are associated with significant carotid epithelial dysfunctions, underlining the fundamental inhibitory role of NO exercised by ET-1^34^. Therefore, the simultaneous presence of CHD from one hand and periodontitis from the other hand, can be a real explanation for the deterioration of endothelial function due to high levels of ET-1. In this regard, recent research has shown that the treatment of periodontitis has significantly reduced the systemic levels of ET-1 in patients with chronic kidney disease^35^.

Moreover, several reports demonstrated that increased hs-CRP levels in serum can facilitate the increment of ET-1 levels in serum in several diseases in humans^23,35–38^. As a support of our results, several shreds of evidence have been shown that situations which may determines an increment of oxidative stress, as CVD and periodontal disease, determines the high release of CRP, which in turn, can arouse the production of ET-1 in saliva and serum in order to defend tissue damage determined by oxidative stress condition^36^. In agreement with the results of the present study, Ekuni *et al*.^39^ demonstrated that ET-1 CRP and ET-1 levels were higher during active phases of periodontal disease.

However, while evidence has previously been demonstrated regarding high serum ET-1 levels as primary mediators of endothelial dysfunction or in the development of cardiovascular risk, from the author’s knowledge, there is no specific evidence to determine ET-1 levels in saliva in order to evaluate whether the increased expression of salivary ET-1 levels determines, by reflection, an increase in ET-1 in serum and then analyzes the salivary levels of ET-1 as an index of endothelial dysfunction. In this regard, however, it should be noted that this study did not reveal a significant correlation between serum and saliva ET-1 levels, as salivary ET-1 levels are influenced in patients enrolled independently only of hs-CRP and total cholesterol. This explanation can be determined by the way that ET-1 salivary levels may be due to an exclusive local oral production of ET-1.

In this regard, it should be noted that, from the studies currently present in the literature, while the effect of ET-1 at a systemic level mediated by the reduction of NO on endothelial damage has been previously highlighted, the impact of ET-1 activation orally (eg salivary) it is however less clear. However, there are studies that show that periodontal disease is positively correlated with high levels of NO and therefore with related stress-oxidative damage^19,24^. The presence of high levels of NO at salivary level can be explained as NO is produced orally in response of the host as a specific salivary defense in the presence of periodontal pathogenic bacteria that are exacerbated during periodontitis^12,24,40,41^. However, there is no unanimous consensus in the literature on the effects of NO levels on tissue damage during periodontitis. Some reports have shown high levels of NO in periodontal tissue in the active periodontal period^39,42^, while, on the other hand, other authors have shown lower levels of NO in saliva of subjects with periodontal disease^25,43^. This discrepancy however, the results in the literature may have been determined by the different homogeneity of the patients enrolled in the studies, by the different age ranges of the patients analyzed or by the excessive presence of enrolled smoking patients; in fact it has been shown that smoking can cause a high increase in salivary NO salivary levels^44^. Another explanation for the different results found in the literature can be determined by the different salivary sampling method in the different studies and by the method used in them to analyze the different markers. Furthermore, the cause of the different expression of ET-1 at the salivary and serum level may be due to a different production of NO at the oral level which may be different from the serum one.

As an explanation of the results of the present study, it should be highlighted that the dysfunctional damage at the endothelium level found in patients with periodontitis and with CHD can be determined by a specific inflammatory and immune pathway in which ET-1 modulates a response towards pathogenic bacteria of the oral biofilm which are exacerbated during the active phases of periodontal damage. It has also been shown that ET-1, during periodontal disease, mediates the immune response at the endothelial level through specific heat shock proteins which has been shown to be useful for stimulating the production of cross-reactive T cells^45–50^. In this regard, this process which sees ET-1 as a key modulator, has also been shown to influence the host defense mechanism that determines a subsequent activation of endothelial cell production which leads to an increased risk of future tissue damage effects due to periodontal pathogens bacteria in several oral diseases^43,47–56^.

However, the present trial has some limitations. Among the main limitations there is the type of study, which makes it difficult to analyze the cause and effect on a temporal level of ET-1. The small sample size, due to high inclusion and exclusion levels, and to the important excluded confounders, also represents a limitation of the present preliminary study. However, the exclusion of several confounders represents a positive and rigorous aspect for the clear evaluation of these confounders on the concentration of serum and salivary levels of ET-1.

Recently, different approaches have been developed with the aim of easily evaluating innovative salivary markers useful for early and subclinically validating the development of different diseases. The results of this study suggest that patients suffering from periodontitis and CHD have higher serum and salivary levels of ET-1 than subjects with periodontitis and healthy controls.

The results of the present study suggests that mainly CHD acts as a stimulus to the increased serum ET-1 levels may be beyond a pathway intermediated by hs-CRP. Therefore, the results of this preliminary study are encouraging but at the same time require further studies with a larger sample of analysis in order to better comprehend the function of ET-1 during periodontitis.

## Supplementary information

Supplementary figure.

Supplementary information.

## Data Availability

The study was performed by the Declaration of Helsinki, revised in 2016 on medical research. Ethical approval was obtained by the IRB of the University of Messina (#16012). The study was registered at clinicaltrials.gov (NCT04143574). Written informed consent was obtained from each patient about the study characteristics and possible risks of the study.
