# Supplementary figures and images for: Analysis of Endothelin-1 Concentrations in Individuals with Periodontitis
Source: Sci Rep. 2020 Feb 3;10:1652. doi: 10.1038/s41598-020-58585-4 (PMC6997234; doi:10.1038/s41598-020-58585-4)

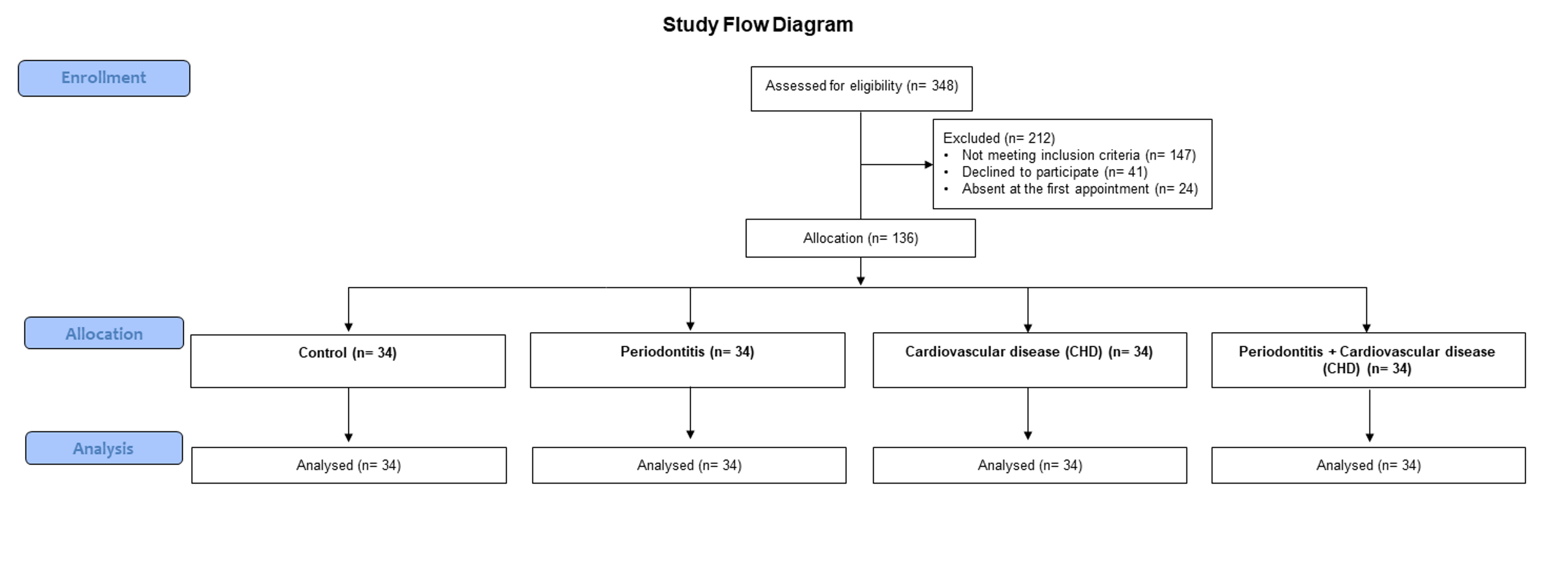

Supplement: Supplementary file 1 — Supplementary figure. [file 41598_2020_58585_MOESM1_ESM.tif]
